# Work-Related Low Back Pain and Psychological Distress Among Physiotherapists in Saudi Arabia: A Cross-Sectional Study

**DOI:** 10.3390/healthcare13151853

**Published:** 2025-07-30

**Authors:** Amjad Abdullah Alsenan, Mohamed K. Seyam, Ghada M. Shawky, Azza M. Atya, Mohamed A. Abdel Ghafar, Shahnaz Hasan

**Affiliations:** 1 Department of Physical Therapy, Comprehensive Rehabilitation Center, Ministry of Human Resources and Social Development, Riyadh 55422, Saudi Arabia; amjadalsenan@hotmail.com; 2 Department of Physical Therapy and Health Rehabilitation, College of Applied Medical Sciences, Majmaah University, Al Majmaah 11952, Saudi Arabia; m.seyam@mu.edu.sa (M.K.S.); gha.shawky@mu.edu.sa (G.M.S.); 3 Rehabilitation Sciences Department, Health and Rehabilitation Sciences College, Princess Nourah bint Abdulrahman University, P.O. Box 84428, Riyadh 11671, Saudi Arabia; amattia@pnu.edu.sa; 4 Physical Therapy Program, Batterjee Medical College, Jeddah 21442, Saudi Arabia; pt12.jed@bmc.edu.sa

**Keywords:** musculoskeletal disorders (MSDs), physiotherapists, low back pain, mental health, Saudi Arabia

## Abstract

**Background**: Musculoskeletal disorders significantly affect healthcare professionals, particularly physiotherapists, due to the physical demands of their work. The link between physical ailments and psychological distress is especially prominent in clinical settings. **Objectives**: To assess the prevalence of work-related low back pain (LBP), stress, anxiety, and depression among physiotherapists in Saudi Arabia, and to identify associated local risk factors. **Methods**: A cross-sectional study using convenience sampling included 710 licensed physiotherapists across Saudi Arabia. Participants completed an online survey containing demographic data and the validated measures, including the Visual Analog Scale (VAS) for pain, the Oswestry Disability Index (ODI), and the Depression, Anxiety, and Stress Scale-21 (DASS-21) for psychological distress. Data were analysed using descriptive statistics, chi-square tests, correlation, and regression analyses. **Results**: Of 710 responses, 697 were valid; 378 physiotherapists reported work-related LBP. The mean pain intensity was 4.6 (SD = 1.6), with 54.2% experiencing moderate to severe disability. Mental health results showed 49.7% had depressive symptoms and 33.9% experienced some level of anxiety. Significant correlations were observed between disability and psychological distress (anxiety: r = 0.382; depression: r = 0.375; stress: r = 0.406; all *p* < 0.001). Regression analyses indicated psychological distress significantly predicted disability, with R^2^ values ranging from 0.125 to 0.248, being higher among inpatient physiotherapists. **Conclusions**: This study reveals a high prevalence of LBP and psychological distress among Saudi physiotherapists, with stress being the strongest predictor of LBP severity. Integrated ergonomic and mental health interventions, including workplace wellness programs and psychological support, are recommended to reduce risks and promote a healthier, more sustainable physiotherapy workforce.

## 1. Introduction

Musculoskeletal disorders (MSDs) are a broad category of conditions that affect the body’s musculoskeletal system, including muscles, bones, joints, ligaments, tendons, and associated structures such as nerves and blood vessels [1]. These disorders commonly arise from repetitive movements, sustained force, vibrations, awkward postures, or poor ergonomic practices, often compounded by previous injuries, lifestyle factors, and underlying health conditions [2]. In occupational settings, when job demands exceed the body’s functional limits, MSDs may develop, frequently leading to pain and functional impairment. The World Health Organization recognizes work-related MSDs as a significant global health burden, especially those affecting the neck, shoulders, back, and upper limbs, which often lead to degenerative or inflammatory complications [1].

Among all types of MSDs, low back pain (LBP) is the most prevalent and disabling condition worldwide, cutting across various professions and geographic boundaries [3]. LBP is responsible for substantial physical discomfort and reduced functional ability, and is one of the leading causes of work absenteeism, disability, and healthcare utilization. It has been identified as a common occupational hazard, particularly in physically demanding professions, including healthcare [4,5]. Physiotherapists, as frontline rehabilitation providers, are uniquely exposed to high physical demands during daily work, such as manual patient handling, repetitive lifting, prolonged static postures, and frequent bending or twisting movements [6]. These biomechanical stressors place them at considerable risk for developing LBP, with studies reporting lifetime prevalence rates ranging from 50% to 80% among physiotherapy professionals [7,8]. LBP among physiotherapists not only affects their personal health and productivity but also has implications for the quality of care they provide. Chronic pain can impair motor performance, reduce motivation, and disrupt clinical routines. Furthermore, the fear of exacerbating pain often leads to work adjustments or limitations in scope of practice, which may compromise patient handling and therapeutic interventions [9].

Global trends reveal variability in the prevalence and impact of LBP among physiotherapists across different regions. While lower rates have been reported in some Asian and American contexts, higher prevalence is observed in African and European countries, likely due to differences in work environments, workload, ergonomics, and reporting practices [3,10]. Despite widespread international evidence, studies specifically focusing on LBP among physiotherapists in Saudi Arabia remain scarce. This presents a critical gap in understanding the regional burden and determinants of LBP in a healthcare system that is currently undergoing substantial transformation. The Kingdom of Saudi Arabia is implementing large-scale reforms in the healthcare sector under Vision 2030, which emphasizes improved service delivery, increased patient access to care, workforce optimization and improvements to rehabilitation infrastructure [11,12]. While these initiatives aim to enhance healthcare outcomes, they may also increase workload, extend service hours, and place administrative demands on healthcare providers, including physiotherapists. There will be greater demand for physiotherapists, potentially increasing physical and psychological strain due to intensified responsibilities and evolving professional expectations in diverse clinical settings. With increased patient loads, pressure to meet performance targets, and expanded rehabilitation roles, physiotherapists may face a heightened risk for work-related injuries and stress-induced health problems. Moreover, the unique cultural, social, and institutional dynamics in Saudi Arabia may influence physiotherapists’ physical health, coping behaviors, and help-seeking tendencies. Therefore, context-specific evidence is essential to guide preventive strategies and policy interventions. Investigating these elements is essential for developing context-specific interventions that cater to the needs of physiotherapists in the region [13].

Similar research indicates that LBP in physiotherapists is not only widespread but also closely linked to modifiable workplace factors such as poor ergonomics, insufficient rest breaks, inadequate equipment, and lack of body mechanics training [9]. Occupational exposure to these risk factors, if unaddressed, leads to cumulative trauma and chronicity of symptoms. Additionally, gender, years of clinical experience, and specific work settings (e.g., inpatient vs. outpatient care) may also influence the prevalence and severity of LBP. Notably, previous studies in the Gulf region suggest that cultural attitudes toward pain, physical activity levels, and stress management practices also play a role in shaping the experience and reporting of LBP [9,14].

Beyond physical implications, LBP has been consistently associated with mental health conditions such as occupational stress, burnout, and depression among healthcare workers [10,15]. In physiotherapists, persistent LBP may compromise their professional identity, reduce self-efficacy, and generate feelings of inadequacy or failure in delivering patient care. These psychological consequences are further aggravated by job insecurity, fear of re-injury, and limited institutional support. Over time, these challenges can result in absenteeism, presentism, and premature exit from the profession. Recognizing and addressing the psychological burden associated with LBP is therefore critical to ensuring a resilient and effective physiotherapy workforce. This biopsychosocial interplay is particularly relevant in physiotherapy settings, where job performance heavily relies on both physical and cognitive engagement.

Given the current gaps in the literature and the evolving nature of healthcare services in Saudi Arabia, a targeted investigation into the prevalence and determinants of work-related LBP among physiotherapists is both timely and necessary. It is essential to explore not only the physical risk factors but also the psychological dimensions of LBP, including stress and depression, as part of a comprehensive occupational health framework. Understanding how local work conditions, cultural norms, and healthcare reforms intersect with physical and mental well-being will offer actionable insights into improving workplace safety and clinician satisfaction. Therefore, this study aims to assess the prevalence of work-related LBP among physiotherapists working in inpatient and outpatient settings across Saudi Arabia, while also examining associated levels of occupational stress and depressive symptoms. The goal is to identify key risk factors that can inform the design of evidence-based interventions, including ergonomic modifications, workload adjustments, mental health support systems, and training in injury prevention.

We hypothesized that the prevalence of work-related LBP among physiotherapists in Saudi Arabia is significantly associated with occupational risk factors such as workload, posture, and years of experience.

We also hypothesized that physiotherapists experiencing LBP will report significantly higher levels of depression, anxiety, and stress than those without LBP. By filling this critical research gap, the study contributes to national efforts to promote healthcare worker safety, aligns with Saudi Arabia’s Vision 2030 goals, and supports the development of a sustainable and healthy physiotherapy workforce.

## 2. Methodology

### 2.1. Research Design

This study adopted a cross-sectional design, focusing on physiotherapists working across Saudi Arabia. This design is suitable for assessing the prevalence and determinants of work-related health issues among this population. The research was approved by the Majmaah University Research Ethics Committee (Ethics number: MUREC-May.14/COM-2024/17-3). Participation was voluntary, and all responses were collected anonymously. We referred to the STROBE guidelines when reporting the observational study design.

### 2.2. Sample Size Estimation

To ensure adequate statistical power for estimating the prevalence of work-related LBP and identifying its psychological correlates among physiotherapists in Saudi Arabia, a priori sample size estimation was conducted using G*Power 3.1. For the primary objective of estimating LBP prevalence with a 95% confidence level and a 5% margin of error, assuming a conservative prevalence estimate of 50%, the minimum required sample size was calculated to be 385 participants. This ensures reliable generalization of prevalence findings across the population of registered physiotherapists in Saudi Arabia. For secondary objectives involving the identification of associations between LBP and psychological factors, the analysis assumed a medium effect size (Cohen’s w = 0.3), alpha of 0.05, and power of 0.80, resulting in a minimum required sample of 88 participants.

### 2.3. Participants

Data were collected using convenience sampling through an online survey. Participants aged 20–65, employed in Saudi Arabia, registered with the Saudi Commission for Health Specialties (SCFHS), and with at least one year of clinical experience were included in this study. Exclusions applied to those with LBP from trauma, interns, students, and non-clinically active physiotherapists. All the participants were informed about the study’s purpose and significance, and written informed consent was obtained before they completed the questionnaires.

### 2.4. Procedure

Ethical approval was obtained from the Institutional Ethical Committee prior to the study’s commencement. The questionnaire was designed and the survey link was distributed via social media and emails to members of the Saudi Physiotherapy Association (SPTA). Data quality was ensured by using mandatory fields, logic checks, and one-response-per-IP restrictions. Incomplete, duplicate, or inconsistent responses were excluded from the final dataset.

### 2.5. Operational Definitions

In this study, mental health issues refer specifically to three distinct constructs—stress, anxiety, and depression—with each representing unique psychological states with clinical and public health relevance. Stress was defined as the body’s response to perceived challenges or threats, leading to emotional and physiological tension [16]. Anxiety was characterized by persistent feelings of worry, fear, or nervousness that are excessive or disproportionate to actual threats (American Psychiatric Association, 2013) [17]. Depression involved a sustained period of low mood, loss of interest in usual activities, and feelings of hopelessness, often accompanied by changes in appetite, sleep, and concentration (WHO, 2020) [18]. Although these constructs are interrelated, they represent separate dimensions of mental health and require distinct approaches to assessment and management. For the purposes of this study, each was considered individually to capture a comprehensive view of participants’ psychological well-being.

### 2.6. Questionnaire

A questionnaire was designed based on the available literature to gather comprehensive information from physiotherapists and consisted of four sections [19,20]. The demographics section included age, nationality, height, weight, marital status, smoking status, chronic diseases, main work setting, subspecialty, licensing status, work hours per week, and experience with LBP. The VAS assessed symptom intensity on a continuum from “no pain” to “worst possible pain,” quantifying work-related LBP. The Oswestry Disability Questionnaire (ODI) evaluated disability due to LBP, while the Depression Anxiety Stress Scales-21 (DASS-21) measured psychological distress [21,22]. The DASS-21 is a 21-item self-report scale measuring psychological distress across three domains: depression, anxiety, and stress, with seven items per domain. Each item is rated on a 4-point Likert scale (0–3), and subscale scores are doubled to obtain final scores ranging from 0 to 42. Severity is categorized as normal, mild, moderate, severe, or extremely severe. Higher scores indicate greater emotional distress. The DASS-21 is a widely validated tool. It is commonly used in clinical and occupational health research to assess mental well-being.

The VAS is a widely recognized tool used to measure the intensity of symptoms, particularly pain [23]. It typically consists of a 10 cm horizontal line marked at each end with descriptors such as “no pain” and “worst possible pain.” In the context of this study, the VAS was utilized to quantify the severity of work-related LBP, stress, and depression among participants. The Arabic version of the ODI is a key outcome measure for assessing disability related to LBP [24]. It evaluates how pain affects daily activities across ten sections, including personal care, lifting, and social life. Each section contains six statements that participants select based on their experiences, with scores ranging from 0 (no disability) to 5 (maximum disability). A score of 30% or higher indicates significant disability, reflecting a moderate to severe impact on daily functioning. ODI demonstrates high internal validity and reliability, making it a valuable tool for this study [21]. The Arabic version of the DASS-21 consists of 21 items that assess emotional states of depression, anxiety, and stress over the past week [25]. Respondents use a 4-point Likert scale to indicate the severity of their experiences. The DASS-21 exhibits high internal consistency with Cronbach’s alpha values typically exceeding 0.80, and good test–retest reliability, making it a reliable measure for tracking emotional states over time [26]. DASS-21 subscales were used both as continuous variables (for regression and correlation analyses) and categorical severity levels (for descriptive statistics and group comparisons), allowing for a comprehensive analysis of psychological distress.

### 2.7. Statistical Analysis

Descriptive statistics (means, standard deviations, frequencies, and percentages) were used to summarize demographic characteristics and survey responses. The normality was assessed using the Shapiro–Wilk test and based on the distribution of the data, Pearson correlation coefficients and simple linear regression analyses were used to assess relationships between continuous variables. Prior to conducting linear regression, key assumptions were assessed to ensure the validity of the models. Linearity was evaluated through scatterplots, and normality of residuals was examined using Q–Q plots, histograms, and the Shapiro–Wilk test. Homoscedasticity was assessed by inspecting residuals-versus-fitted value plots and confirmed using the Breusch–Pagan test. Independence of errors was tested using the Durbin–Watson statistic, and multicollinearity was checked through variance inflation factors. Chi-square tests were performed to examine associations between categorical variables. For comparisons between groups with non-normally distributed data, the Mann–Whitney U test was employed. A two-tailed *p*-value of <0.05 was considered statistically significant for all analyses. All statistical analyses were conducted using SPSS version 21.

## 3. Results

### 3.1. Sample Characteristics

**Figure 1:**
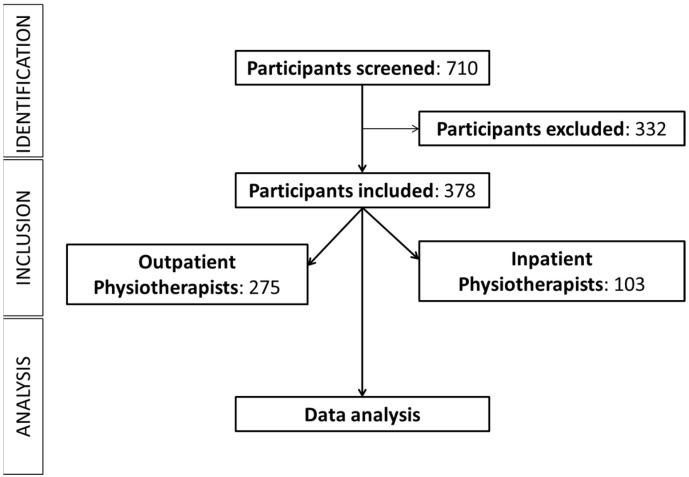
STROBE-adapted flow chart.

A total of 712 responses were collected through the online questionnaire. After excluding incomplete responses and participants without valid licensing from the Saudi Commission for Health Specialties, the final sample comprised 697 physiotherapists. Of these, 378 participants reported experiencing work-related LBP and were included in the current analysis. Details are presented in the STROBE-adapted flow chart (Figure 1).

The sample was predominantly female and young, with a mean age in the late twenties. Most participants had a bachelor’s degree in physical therapy and reported no chronic diseases. The majority were non-smokers and Saudi nationals, with more than half working in outpatient departments. Notably, a large proportion worked more than 35 h per week, reflecting high professional engagement. Further demographic details are presented in Table 1.

### 3.2. Pain Intensity and Disability

**Figure 2:**
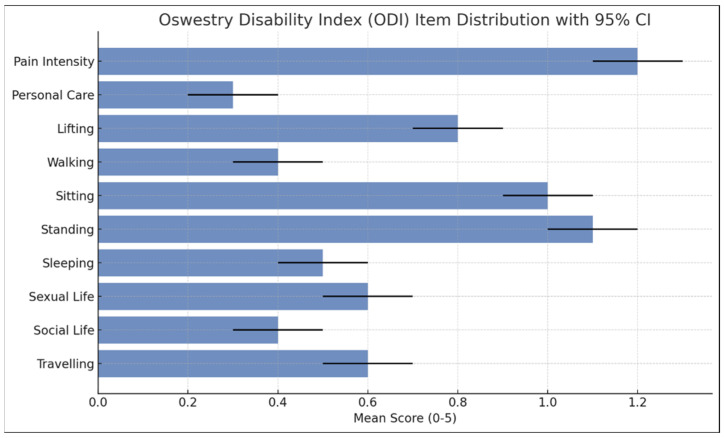
Mean ODI item scores with 95% confidence intervals (n = 378). Higher scores reflect greater functional disability per domain.

Participants reported moderate pain levels on the VAS. Disability levels, assessed via the ODI, indicated that the majority experienced minimal disability, while a smaller subset reported moderate limitations in daily activities. Only a few participants reported severe disability, and none were classified as ‘crippled.’ The highest disability ratings were related to pain intensity, standing, and sitting. Detailed scores are provided in Table 2 and Figure 2.

### 3.3. Mental Health Assessment

Psychological distress was evaluated using the DASS-21. Around half of the participants reported symptoms of depression or anxiety, while the majority reported normal stress levels. However, there was considerable variation in severity across the sample, with some individuals experiencing severe or extremely severe symptoms in all three domains. The mean DASS-21 scores suggested mild depression, moderate anxiety, and normal stress levels overall. Full distributions are shown in Table 3.

### 3.4. LBP, Psychological Distress, and Disability by Workplace Setting

Comparisons between outpatient and inpatient physiotherapists revealed no statistically significant differences in the prevalence of LBP, anxiety, depression, or stress. However, inpatient physiotherapists reported significantly higher disability scores, indicating a greater functional impact of LBP in this group (*p* = 0.029). Further group comparisons are summarized in Table 4.

### 3.5. Correlations Between Disability and Psychological Distress

**Figure 3:**
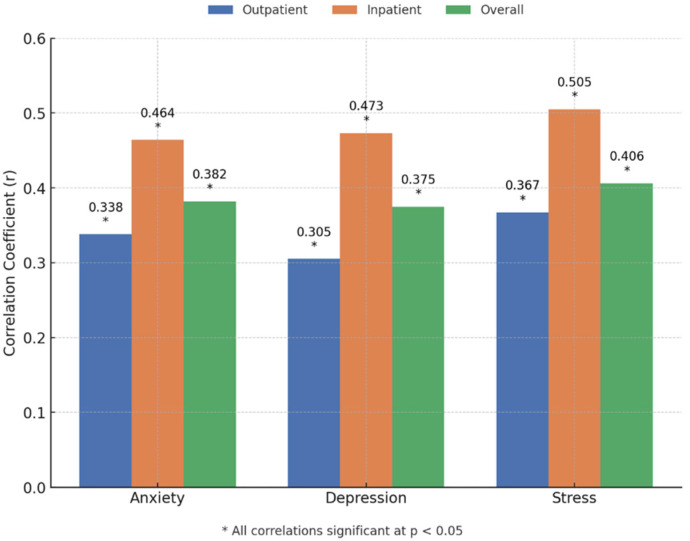
Correlation coefficients between disability and psychological distress variables (anxiety, depression, and stress) in outpatient, inpatient, and overall samples. All correlations are statistically significant (*p* < 0.05).

Positive correlations were observed between disability and psychological distress variables, including anxiety, depression, and stress. These relationships were particularly pronounced in inpatient physiotherapists, suggesting that psychological factors may have a stronger association with functional limitations in this subgroup. The outpatient group also showed significant, though slightly weaker, correlations. Details about correlation are provided in Figure 3.

### 3.6. Psychological Distress as Predictors of Low Back Pain

Regression analyses demonstrated that anxiety, depression, and stress were significant predictors of LBP severity in the overall sample, accounting for 12% to 14% of the variance when examined separately. Stratified analyses indicated stronger predictive relationships in inpatient physiotherapists compared to those in outpatient settings, suggesting a heightened psychological impact in more physically demanding work environments.

The multivariate regression model, which included all three psychological variables alongside age, BMI, and years of experience, explained 29.3% of the variance in LBP severity for inpatient physiotherapists, 17.8% for outpatient physiotherapists, and 19.8% for the overall sample. These findings emphasize the combined influence of psychological distress and demographic factors on LBP among physiotherapists. Full results are presented in Table 5.

### 3.7. Work-Related Factors and LBP

Analyses did not reveal any significant associations between specific work-related factors (such as working hours or clinical setting) and the presence of LBP, anxiety, depression, or stress. This suggests that individual psychological and physical health factors may play a more critical role than work environment alone. Additional details are presented in Table 6.

## 4. Discussion

This study provides a comprehensive examination of the prevalence of LBP and mental health issues among physical therapists in Saudi Arabia, emphasizing the urgent need for targeted interventions. The findings reveal that 54.2% of the surveyed physiotherapists experience work-related LBP, with a significant proportion also reporting symptoms of anxiety, depression, and stress. These results align with the existing literature, highlighting the multifaceted nature of the health challenges faced by health care professionals and the critical intersection of physical and mental well-being. The final sample of 697 physiotherapists, including 378 currently reporting work-related LBP, far exceeds the minimum required thresholds for both prevalence estimation and inferential analyses, thus providing high confidence in the reliability and statistical power of the study’s findings.

The study’s sample of 710 physical therapists, of whom 378 reported LBP, predominantly consisted of younger, female Saudi nationals with a mean age of 29.7 years. This demographic aligns with earlier findings which noted similar trends in occupational health among healthcare providers [5]. The prevalence of LBP, particularly among those working in inpatient settings, underscores the occupational hazards inherent in this profession. Notably, while the majority of respondents exhibited minimal disability, a concerning proportion reported moderate levels of distress linked to their mental health. The association between LBP and psychological distress is particularly alarming, as it suggests that mental health factors could be strong predictors not only of LBP but also of overall job satisfaction and performance. Our findings are consistent with those of Abdelaziz et al. (2024), who reported significant rates of anxiety and stress in patients with LBP, reinforcing the need to consider psychological assessments in both clinical and occupational settings [27].

The study’s findings resonate with previous research. Bae and Min (2016) reported a high prevalence of work-related MSDs among physiotherapists in South Korea, emphasizing the global nature of this issue [28]. However, significant differences exist in the predictors of LBP across studies. While Bae and Min identified workplace factors such as hours worked and experience as significant, our study found no such associations. This discrepancy may reflect cultural differences in workplace expectations or variations in healthcare systems. In a similar vein, Alharbi [13] highlighted the prevalence of LBP among nurses in Saudi Arabia, further supporting the notion that healthcare professions are particularly vulnerable to musculoskeletal disorders. However, our study extends the analysis to include mental health predictors, suggesting a more comprehensive understanding of the factors affecting physiotherapists’ well-being.

The study by Haris et al. (2024) examined 100 physiotherapists in Karachi, contrasting with a larger current study involving 697 participants, which highlighted stress, anxiety, and depression as predictors of LBP [29]. While Haris et al. found a weak link between stress and physical ability, the current study indicated a stronger correlation, possibly due to its focus on LBP rather than general physical ability. Mansour et al. (2022) reported a high prevalence of work-related MSDs among 139 physiotherapists in Jordan, noting no significant relationship with depression, unlike the current study, which found a considerable correlation between mental health and LBP disability [30]. Mikołajewski et al. (2023) compared physiotherapists to IT professionals in Poland, revealing higher stress levels in the former but differing in findings related to age and tenure [31]. The development of work-related musculoskeletal pain among IT professionals in Saudi Arabia has been shown to affect their activities of daily living [32]. Woodman et al. (2022) provided a broader context, indicating a 70.5% prevalence of LBP in Saudi healthcare, reinforcing the occupational risks [33]. These studies highlight the complex interplay between mental health, LBP, and workplace factors, suggesting that psychological factors may outweigh structural ones in influencing health outcomes, underscoring the need for targeted psychological interventions in occupational health [32,33]. Since the higher prevalence of work-related MSDs among healthcare professionals in Saudi Arabia has been shown to affect their daily activities, even forcing them to change their work setting, further studies should emphasize the role of ergonomics, counselling, proper techniques of patient handling, etc., during training [5,9,20].

The findings of this study also demonstrate significant predictive relationships between anxiety, depression, and stress and LBP among physical therapists, further emphasizing the importance of mental health in occupational health contexts. Specifically, the inpatient department exhibited a stronger association among these psychological factors compared to outpatient settings, aligning with findings from previous studies that highlight the impact of workplace stressors on physical health [30,33]. Conversely, no significant correlations were found between work-related factors and LBP or mental health, suggesting that psychological factors may play a more crucial role than occupational conditions in this population [29,31]. These findings underscore the need for integrated mental health interventions in workplace health programs to address the psychological dimensions affecting LBP among physical therapists.

The findings of this study, while being specific to physiotherapists in Saudi Arabia, may have broader applicability to other countries with similar healthcare systems, cultural dynamics, and workforce demands. In nations undergoing rapid healthcare expansion, like such as other Gulf countries, the increased workload, understaffing, and evolving healthcare infrastructure mirror the challenges faced in Saudi Arabia. Additionally, cultural norms regarding work expectations and help-seeking behavior may contribute similarly to the physical and psychological burden on healthcare workers. Therefore, while caution is warranted in generalizing these results globally, they are likely relevant to physiotherapy populations in comparable Middle Eastern and developing healthcare settings, where systemic occupational health measures are still evolving.

This study provides one of the first large-scale analyses of work-related LBP and its psychological correlates among physiotherapists in Saudi Arabia, a region where such data are scarce. Unlike prior global research, which often focuses solely on prevalence, this study explores the interplay between disability, anxiety, depression, and stress, while also comparing inpatient and outpatient settings. By integrating both physical and psychological factors in a rapidly evolving healthcare landscape under Saudi Arabia’s Vision 2030 reforms, this research offers culturally relevant insights that can inform tailored interventions. These findings not only fill a critical regional gap but also contribute to the broader global understanding of occupational health challenges faced by physiotherapists in diverse healthcare systems. While individual strategies such as ergonomic awareness and self-care are important, this study emphasizes the need for institutional-level interventions. Healthcare organizations should implement comprehensive occupational health policies, including ergonomic workplace assessments, workload management, mental health support services, and regular training programs. Such system-wide initiatives are crucial to sustainably reduce the burden of LBP and psychological distress among physiotherapists, ensuring long-term workforce health and patient care quality.

### Limitations and Recommendations for Future Studies

This study presents several limitations that must be acknowledged. The cross-sectional design limits the ability to infer causality between LBP and mental health conditions, highlighting the need for longitudinal research to understand their temporal relationships better. Additionally, the reliance on self-reported data introduces potential biases, such as recall and social desirability biases, which could affect symptom accuracy. The absence of a control group restricts the generalizability of the findings, and the geographic and demographic focus means that the results may not apply to physiotherapists in different healthcare contexts or regions. Although key confounders such as comorbidities and BMI were controlled for, specific ergonomic factors were not assessed, representing a study limitation. To enhance future research, several recommendations are proposed. First, longitudinal studies should be conducted to explore the causal relationships between LBP and mental health over time. Including more diverse samples of healthcare professionals would improve generalizability and enable cross-cultural comparisons. Incorporating objective measures, such as physiological markers of stress, would strengthen the research’s validity. Additionally, intervention studies evaluating ergonomic training and psychological support initiatives could provide valuable insights into effective strategies for mitigating LBP and enhancing mental health. Qualitative research exploring the lived experiences of physiotherapists could reveal nuanced insights that quantitative measures might miss. As factors like age, BMI, and work experience are known to influence both psychological and musculoskeletal symptoms, including a multivariate regression model in further studies will significantly strengthen the analysis by adjusting for key potential confounders.

## 5. Conclusions

In conclusion, this study highlights the significant prevalence of LBP and associated mental health concerns among physiotherapists in Saudi Arabia. The findings emphasize the critical role of psychological distress, particularly stress, followed by depression and anxiety, in predicting LBP severity, especially in inpatient settings where physical and emotional demands are higher. These results underscore the need for comprehensive interventions that address both ergonomic and mental health factors. Institutions should prioritize workplace wellness programs, provide mental health support, and implement ergonomic training to reduce the risk of LBP and its psychological impact. Promoting work–life balance through flexible scheduling, introducing institutional wellness initiatives, and integrating both physical rehabilitation and psychological support can foster a healthier work environment. Ongoing research and systematic monitoring are also recommended to track progress and evaluate the effectiveness of these interventions. By adopting these strategies, healthcare organizations can enhance physiotherapists’ health and productivity, ultimately contributing to a more sustainable and resilient healthcare system in Saudi Arabia. Further longitudinal research is warranted to explore causal relationships and develop targeted strategies to enhance physiotherapist’s health and professional sustainability.

## Figures and Tables

**Table 1:** Demographic characteristics of the participants (n = 378): values are presented as n (%).

Characteristic	n = 378
**Gender**	
Female	241 (63.8%)
Male	137 (36.2%)
**Nationality**	
Saudi	357 (94.4%)
Other	21 (5.6%)
**Marital Status**	
Unmarried	241 (63.8%)
Married	125 (33.1%)
Divorced	10 (2.6%)
Widowed	2 (0.5%)
**BMI**	
Normal (18.5–24.9)	161 (42.6%)
Overweight (25–29.9)	123 (32.5%)
Obese (>30)	79 (20.9%)
Thin (<18.5)	15 (4.0%)
**Chronic Diseases**	
Yes	44 (11.6%)
No	334 (88.4%)
**Workplace Type**	
Outpatient	275 (72.8%)
Inpatient	103 (27.2%)
**Years of Experience**	
1–5 years	253 (66.9%)
>5–10 years	88 (23.3%)
>10 years	37 (9.8%)
**Duration of Pain**	
Up to 3 days	44 (11.6%)
>3 days but <3 months	101 (26.7%)
>3 months but <1 year	79 (20.9%)
>1 year	154 (40.7%)

**Table 2:** Participants’ pain intensity (VAS, 0–10 cm) and disability levels (Oswestry Disability Index scores). ODI items scored from 0 (no disability) to 5 (maximum disability). Overall ODI percentage calculated by summing item scores, dividing by total possible score, and multiplying by 100.

Measure	Mean (SD)	95% CI
**Pain Intensity (cm)**		
VAS Score (0–10)	4.6 (1.6)	4.4–4.8
**Oswestry Disability Index (ODI, score 0–5)**		
Pain Intensity	1.2 (0.8)	1.1–1.3
Personal Care	0.3 (0.5)	0.2–0.4
Lifting	0.8 (0.9)	0.7–0.9
Walking	0.4 (0.6)	0.3–0.5
Sitting	1.0 (0.8)	0.9–1.1
Standing	1.1 (0.9)	1.0–1.2
Sleeping	0.5 (0.6)	0.4–0.6
Sexual Life	0.6 (0.8)	0.5–0.7
Social Life	0.4 (0.7)	0.3–0.5
Travelling	0.6 (0.7)	0.5–0.7
Overall ODI Score (score 0–50)	6.8 (4.7)	6.3–7.3
Disability (%)	14.4%	12.6–16.2%

**Table 3:** Mental health assessment of participants using the Depression Anxiety Stress Scales-21 (DASS-21) (n = 378) with three subscales: Depression, Anxiety and Stress. The “Interpretation” column categorizes participants’ scores into severity levels based on DASS-21 standard cut-offs.

Condition (n = 378)	n (%)	Mean Score (SD)	Severity Interpretation
**Depression**			
**Not Depressed**	190 (50.3%)	10.74 (9.66)	Mild
**Mild**	52 (13.8%)		
**Moderate**	80 (21.2%)		
**Severe**	28 (7.4%)		
**Extremely Severe**	28 (7.4%)		
**Anxiety**			
**Normal**	183 (48.4%)	9.57 (8.81)	Moderate
**Mild**	33 (8.7%)		
**Moderate**	77 (20.4%)		
**Severe**	33 (8.7%)		
**Extremely Severe**	52 (13.8%)		
**Stress**			
**Normal**	250 (66.1%)	12.87 (9.65)	Normal
**Mild**	41 (10.8%)		
**Moderate**	41 (10.8%)		
**Severe**	28 (7.4%)		
**Extremely Severe**	18 (4.8%)		

**Table 4:** Prevalence of low back pain, disability, anxiety, depression and stress according to participant’s place of work (n = 378).

(a) Prevalence of Low Back Pain by Workplace Setting (outpatient vs. inpatient).					
**Workplace Setting**	**No Pain n (%)**	**Pain n (%)**	**Total n (%)**	**χ^2^ (1, N = 697)**	***p*** **-Value**
**Outpatient**	245 (47.1%)	275 (52.9%)	520 (74.6%)	1.499	0.221
**Inpatient**	74 (41.8%)	103 (58.2%)	177 (25.4%)		
**Total**	319 (45.8%)	378 (54.2%)	697 (100%)		
(b) Disability Scores by Workplace Setting (outpatient vs. inpatient).					
**Workplace Setting**	**n (%)**	**Mann-Whitney U**		***p*-Value**	
**Outpatient**	269 (73%)	−2.180		0.029	
**Inpatient**	99 (27%)				
**Total**	368 (100%)				
(c) Anxiety Severity by Workplace Setting (outpatient vs. inpatient).					
**Severity Level**	**Outpatient n (%)**	**Inpatient n (%)**	**χ^2^ (4, N = 378)**	***p*-Value**	
**Normal**	133 (48.3%)	50 (48.5%)	8.506	0.075	
**Mild**	25 (9.0%)	8 (7.7%)			
**Moderate**	60 (21.8%)	17 (16.5%)			
**Severe**	27 (9.8%)	6 (5.8%)			
**Extremely Severe**	30 (10.9%)	22 (21.3%)			
**Total**	275 (100%)	103 (100%)			
(d) Depression Severity by Workplace Setting (outpatient vs. inpatient).					
**Severity Level**	**Outpatient n (%)**	**Inpatient n (%)**	**χ^2^ (4, N = 378)**	***p*-Value**	
**Normal**	145 (52.7%)	45 (43.6%)	6.325	0.176	
**Mild**	40 (14.5%)	12 (11.6%)			
**Moderate**	50 (18.1%)	30 (29.1%)			
**Severe**	19 (6.9%)	9 (8.7%)			
**Extremely Severe**	21 (7.6%)	7 (6.8%)			
**Total**	275 (100%)	103 (100%)			
(e) Stress Severity by Workplace Setting (outpatient vs. inpatient).					
**Severity Level**	**Outpatient n (%)**	**Inpatient n (%)**	**χ^2^ (4, N = 378)**	***p*-Value**	
**Normal**	185 (67.2%)	65 (63.1%)	3.119	0.538	
**Mild**	28 (10.1%)	13 (12.6%)			
**Moderate**	26 (9.4%)	15 (14.5%)			
**Severe**	22 (8.0%)	6 (5.8%)			
**Extremely Severe**	14 (5.1%)	4 (3.8%)			
**Total**	275 (100%)	103 (100%)			

**Table 5:** Univariate and multivariate linear regression analyses predicting low back pain severity from psychological distress and demographic variables.

Model	R^2^	F-Value	*p*-Value
**Inpatient Department (n = 103)**			
**Anxiety (Univariate)**	0.207	27.685	<0.001
**Depression (Univariate)**	0.216	29.048	<0.001
**Stress (Univariate)**	0.248	34.575	<0.001
**Multivariate Model (Anxiety + Depression + Stress + Age + BMI + Experience)**	0.293	10.883	<0.001
**Outpatient Department (n = 275)**			
Anxiety **(Univariate)**	0.111	35.254	<0.001
Depression **(Univariate)**	0.090	28.035	<0.001
Stress **(Univariate)**	0.131	42.457	<0.001
**Multivariate Model (Anxiety + Depression + Stress + Age + BMI + Experience)**	0.178	11.302	<0.001
**Overall Sample (n = 378)**			
**Anxiety (Univariate)**	0.143	64.064	<0.001
**Depression (Univariate)**	0.125	54.882	<0.001
**Stress (Univariate)**	0.132	74.106	<0.001
**Multivariate Model (Anxiety + Depression + Stress + Age + BMI + Experience)**	0.198	15.842	<0.001

Note: Univariate models include one predictor each. Multivariate models include anxiety, depression, and stress simultaneously, controlling for age, BMI, and years of experience. All models are significant at *p* < 0.001.

**Table 6:** Associations between work-related factors and the presence of low back pain.

Work-Related Factor (n = 378)	Chi-Square	*p*-Value
**Department Type**	χ^2^ (2, N = 378) = 1.582	0.453
**Years of Experience**	χ^2^ (4, N = 378) = 2.562	0.634
**Work Hours**	χ^2^ (14, N = 378) = 7.045	0.933

## Data Availability

The original contributions presented in this study are included in the article. For further inquiries, please contact the corresponding authors.

## References

[1] Major M.-E., Vézina N. (2015). Analysis of worker strategies: A comprehensive understanding for the prevention of work related musculoskeletal disorders. Int. J. Ind. Ergon..

[2] Da Costa B.R., Vieira E.R. (2010). Risk factors for work-related musculoskeletal disorders: A systematic review of recent longitudinal studies. Am. J. Ind. Med..

[3] Louw Q.A., Morris L.D., Grimmer-Somers K. (2007). The prevalence of low back pain in Africa: A systematic review. BMC Musculoskelet. Disord..

[4] Bork B.E., Cook T.M., Rosecrance J.C., Engelhardt K.A., Thomason M.-E.J., Wauford I.J., Worley R.K. (1996). Work-related musculoskeletal disorders among physical therapists. Phys. Ther..

[5] Alghadir A., Zafar H., Iqbal Z.A. (2015). Work-related musculoskeletal disorders among dental professionals in Saudi Arabia. J. Phys. Ther. Sci..

[6] Gorce P., Jacquier-Bret J. (2023). Global prevalence of musculoskeletal disorders among physiotherapists: A systematic review and meta-analysis. BMC Musculoskelet. Disord..

[7] Lunde L.-K. (2017). Physical Demands at Work: Objectively Measured Exposure and Musculoskeletal Pain in Constructionand Healthcare Workers. Thesis.

[8] Vieira E.R., Schneider P., Guidera C., Gadotti I.C., Brunt D. (2016). Work-related musculoskeletal disorders among physical therapists: A systematic review. J. Back Musculoskelet. Rehabil..

[9] Alghadir A., Zafar H., Iqbal Z.A., Al-Eisa E. (2017). Work-related low back pain among physical therapists in Riyadh, Saudi Arabia. Workplace Health Saf..

[10] Trinkoff A.M., Lipscomb J.A., Geiger-Brown J., Storr C.L., Brady B.A. (2003). Perceived physical demands and reported musculoskeletal problems in registered nurses. Am. J. Prev. Med..

[11] Suleiman A.K., Ming L.C. (2025). Transforming healthcare: Saudi Arabia’s vision 2030 healthcare model. J. Pharm. Policy Pract..

[12] Chowdhury S., Mok D., Leenen L. (2021). Transformation of health care and the new model of care in Saudi Arabia: Kingdom’s Vision 2030. J. Med. Life.

[13] Alshehri M.A., Alhasan H., Alayat M., Al-Subahi M., Yaseen K., Ismail A., Tobaigy A., Almalki O., Alqahtani A., Fallata B. (2018). Factors affecting the extent of utilization of physiotherapy services by physicians in Saudi Arabia. J. Phys. Ther. Sci..

[14] Sikiru L., Hanifa S. (2010). Prevalence and risk factors of low back pain among nurses in a typical Nigerian hospital. Afr. Health Sci..

[15] Andersen L.L., Clausen T., Persson R., Holtermann A. (2013). Perceived physical exertion during healthcare work and risk of chronic pain in different body regions: Prospective cohort study. Int. Arch. Occup. Environ. Health.

[16] Folkman S. (2013). Stress: Appraisal and Coping. Encyclopedia of Behavioral Medicine.

[17] American Psychiatric Association (2013). Diagnostic and Statistical Manual of Mental Disorders.

[18] World Health Organization (2017). Depression and Other Common Mental Disorders: Global Health Estimates.

[19] Mohammed O., Alzahrani H., Marouf E., Shaheen R. (2025). Physiotherapists’ perspectives on the implementation of direct access to physiotherapy services in Saudi Arabia: A cross-sectional study. BMJ Open.

[20] Iqbal Z., Alghadir A. (2015). Prevalence of work-related musculoskeletal disorders among physical therapists. Med. Pracy. Work. Health Saf..

[21] Fairbank J.C. (2014). Oswestry disability index. J. Neurosurg. Spine.

[22] Lee D. (2019). The convergent, discriminant, and nomological validity of the Depression Anxiety Stress Scales-21 (DASS-21). J. Affect. Disord..

[23] Bijur P.E., Silver W., Gallagher E.J. (2001). Reliability of the visual analog scale for measurement of acute pain. Acad. Emerg. Med. Off. J. Soc. Acad. Emerg. Med..

[24] Alnahdi A.H. (2025). The Arabic Oswestry Disability Index as a Unidimensional Measure: Confirmatory Factor Analysis. Spine.

[25] Ali A.M., Ahmed A., Sharaf A., Kawakami N., Abdeldayem S.M., Green J. (2017). The Arabic version of the depression anxiety stress scale-21: Cumulative scaling and discriminant-validation testing. Asian J. Psychiatry.

[26] Chen I.H., Chen C.Y., Liao X.L., Chen X.M., Zheng X., Tsai Y.C., Lin C.Y., Griffiths M.D., Pakpour A.H. (2003). Psychometric properties of the Depression, Anxiety, and Stress Scale (DASS-21) among different Chinese populations: A cross-sectional and longitudinal analysis. Acta Psychol..

[27] Abdelaziz R.A., AbdElnabi H.A., Ali M.M., Elsayed N.M. (2024). Prevalence of Musculoskeletal Problems and Its Relation to Psychological Distress among General Secondary Students Subjected to Online Learning. Zagazig Nurs. J..

[28] Bae Y.-H., Min K.S. (2016). Associations between work-related musculoskeletal disorders, quality of life, and workplace stress in physical therapists. Ind. Health.

[29] Haris M., Bangash N.S., Badar S., Raza K.F., Lata P., Mehreen S., Baqir S.R. (2024). Consequences of Stress on the Workability of Physical Therapists: Stress and Workability of Physical Therapists. THERAPIST (J. Ther. Rehabil. Sci.).

[30] Mansour Z.M., Albatayneh R., Al-Sharman A. (2022). Work-related musculoskeletal disorders among Jordanian physiotherapists: Prevalence and risk factors. Work.

[31] Mikołajewski D., Masiak J., Mikołajewska E. (2023). Selected determinants of occupational stress and burnout in physiotherapists and IT professionals. J. Educ. Health Sport.

[32] Alghadir A.H., Khalid S., Iqbal Z.A. (2022). Work-related musculoskeletal disorders among information technology professionals in Riyadh, Saudi Arabia. Med. Pracy. Work. Health Saf..

[33] Woodman A., Homan M., Niaz A., Al-Jamea L., Akhtar M., Sager M. (2020). Low back pain among healthcare personnel in Saudi Arabia: A systematic review. Ibnosina J. Med. Biomed. Sci..

